# The cascade of hypertension care in Cambodia: evidence from a cross-sectional population-based survey

**DOI:** 10.1186/s12913-022-08232-7

**Published:** 2022-06-29

**Authors:** Savina Chham, Veerle Buffel, Josefien Van Olmen, Srean Chhim, Por Ir, Edwin Wouters

**Affiliations:** 1 National Institute of Public Health, Lot 80, Street 566 & Corner with 289, St 566, Phnom Penh, Cambodia; 2 Centre for Population, Family and Health, Department of Social Sciences, University of Antwerp, Antwerp, Belgium; 3 Department of Family Medicine and Population Health (FAMPOP), Faculty of Medicine and Health Sciences, University of Antwerp, Antwerp, Belgium; 4 Centre for Health Systems Research and Development, University of the Free State, Bloemfontein, South Africa

**Keywords:** Hypertension, Cascade of Care, Health System Intervention, Dropping out, Cambodia, Health Equities, Care Continuum

## Abstract

**Background:**

Hypertension (HTN) is a leading cause of cardiovascular diseases and deaths globally. To respond to the high HTN prevalence (23.5% among adults aged 40–69 years in 2016) in Cambodia, the government (and donors) established innovative interventions to improve access to screening, care, and treatment at different public health system and community levels. We assessed the effectiveness of these interventions and resulting health outcomes through a cascade of HTN care and explored key determinants.

**Methods:**

We performed a population-based survey among 5070 individuals aged ≥ 40 years to generate a cascade of HTN care in Cambodia. The cascade, built with conditional approach, shows the patients’ flow in the health system and where they are lost (dropped out) along the steps: (i) prevalence, (ii) screening, (iii) diagnosis, (iv) treatment in the last twelve months, (v) treatment in the last three months, and (vi) HTN being under control. The profile of people dropping out from each bar of the cascade was determined by multivariate logistic regression.

**Results:**

The prevalence of HTN (i) among study participants was 35.2%, of which 81.91% had their blood pressure (BP) measured in the last three years (ii). Over 63.72% of those screened were diagnosed by healthcare professionals as hypertensive patients (iii). Among these, 56.19% received treatment in the last twelve months (iv) and 54.26% received follow-up treatment in the last three months (v). Only 35.8% of treated people had their BP under control (vi). Males, those aged ≥ 40 years, and from poorer households had lower odds to receive screening, diagnosis, and treatment. Lower odds to have their BP under-control were found in males, those from poor and rich quintiles, having HTN < five years, and receiving treatment at a private facility.

**Conclusions:**

Overall, people with HTN are lost along the cascade, suggesting limited access to appropriate screening, diagnosis, and treatment and resulting poor health outcomes, especially among those who are male, aged 40–49 years, from poorer households, and visiting a private facility. Efforts to improve the quality of facility-based and community-based interventions are needed to prevent inequitable drops along the cascade of care.

## Background

Hypertension (HTN) is a leading cause of cardiovascular diseases (CVD) and deaths globally [1]. An estimated 1.28 billion adults aged 30–79 years live with HTN globally, with two-thirds living in low- and middle-income countries [2]. It is a rising problem in many low- and middle-income countries [3], including Cambodia. The prevalence of HTN among adults aged 40–69 years in Cambodia was 23.5% in 2016 [4].

In response to this high prevalence, Cambodia has been re-organizing its health system to play an essential role in ensuring the effective management of HTN. In 2007, the government included management of HTN as basic care in the minimum package of activities by enabling primary care level facilities, such as health centers (HCs), to treat mild HTN and refer severe and complicated cases to district referral hospitals (RHs) [5]. Through support from the government and donor organizations, additional and innovative interventions for HTN and other non-communicable diseases, especially diabetes, have been introduced at different levels in the public health system and community in selected provinces. These include the following interventions: (1) the establishment of Non-Communicable Disease (NCD) clinics at the RHs, (2) the introduction of the World Health Organization Package of Essential Non-Communicable Disease Interventions (WHO PEN) program in HCs, and (3) the expansion and integration of the community-based MoPoTsyo’s Peer Educator Network.

NCD clinics have been established by the Ministry of Health (MoH) to provide hospital-based care for NCDs at RHs since 2007 with the aim of providing care for people with type 2 diabetes (T2D) and/or HTN through screening, diagnosis, treatment, and health education. Currently, 31 clinics are functional in selected operational districts (ODs) in 17 provinces. With regard to HTN, the NCD clinics offer consultation and treatment services to people living in the area.

The WHO PEN program has been implemented as health center-based care since 2015. This intervention aims to complement the function of the NCD clinics through screening suspected T2D and HTN cases. With regard to HTN, WHO PEN provides counseling, screening, and treatment for mild cases of HTN, and follow-up treatment for referred HTN patients at the HC level. The WHO PEN program has encouraged CVD risk screening, including blood pressure (BP) measurement, for adults aged 40 years and older. It is supported by the WHO and other non-government organizations [6]. As of February 2021, it covered 121 HCs. The activities mentioned in the WHO PEN comply with the new Minimum Package of Activities of the MoH, which allowed the treatment for mild HTN at HCs and referred severe and complicated HTN to RHs [7]. Mild or non-complicated cases of hypertension are allowed to be treated at the HC by mostly secondary nurses and sometimes midwives (with 3 years of pre-service training), and exceptionally by medical doctors in HCs where they are present.

The MoPoTsyo Peer Educator Network, established and supported by a local non-government organization, provides community-based care and support for people with T2D and HTN. In the network, peer educators who are diabetic patients themselves, after being trained by the organization, provide regular follow-up checks and counseling to other patients registered in the network and assist them in getting access to medical services at RHs. With regard to HTN, peer educator networks also organize (1) community-based screening, (2) self-management support, (3) medication supply under a revolving drug fund, and (4) service operation through public facilities. MoPoTsyo is active in 20 districts in 8 provinces [8, 9].

While the interventions above take place at public health facilities, it must be noted that a significant proportion of the population does not use public healthcare [10]. Private facilities have been the primary choice of care for Cambodians when they get sick or injured; evidently, more than 70% of all healthcare visits took place at private healthcare providers in 2016 [11]. Furthermore, a majority (59%) of people with chronic diseases were diagnosed and treated in private facilities [12].

A multitude of interventions exists at both public and private facilities to diagnose and treat HTN in Cambodia, including the aforementioned innovative interventions in the public sector. However, little is known about the outcomes of these interventions. Studying the outcomes of a chronic condition such as HTN is complex since effective care for HTN requires a continuum of care (prevalence, screening, diagnosis, treatment, follow-up treatment, and control of HTN). An adverse health outcome at the end can be caused by a health system weakness at any of these steps. Therefore, applying a Cascade of Care (CoC) approach is critical to assess these outcomes. A CoC is a model to outline the sequential stage of long-term care (prevalence, screening, diagnosis, treatment, follow-up treatment, and control) required to achieve a desirable outcome. From a healthcare system perspective, a CoC model is a very useful tool for understanding where the health system has failed, to quantify losses and identify the characteristics of people lost in each stage of the care continuum.

A first but not comprehensive attempt to assess a cascade of HTN care was made through the national STEPS survey in 2016. This survey showed that, among respondents aged 18–69 years, 12.9% had their BP measured and were diagnosed with HTN. Of these, 32.7% were then taking prescribed medication, while only 16.1% of those on medication had their BP controlled [4]. The 2016 STEPS survey, however, produced the CoC for the population aged 18–69 years, rendering these results less useful to assess outcomes of the health system interventions that targeted the at-risk population. The healthcare guidelines on screening and treatment defined those aged 40 years or older as the ‘at-risk population’. In addition, the 2016 STEPS survey also did not collect additional information on the healthcare use related to HTN from one of the above-cited health system interventions. These limitations render additional up-to-date research on the at-risk population, their healthcare use, and resulting health outcome.

To better understand the effects of the above-cited health system interventions, it is also relevant to look at patient-related factors that influence the outcomes of HTN care. Socio-demographic and economic characteristics, for instance, might impact access to screening, diagnosis, or treatment, and consequently health outcomes in different ways. The influence of such factors on different steps in the cascade is not well documented and might be context-specific. Previous studies using the CoC approach have been previously published in the USA [13, 14], Brazil [15], and other Asian countries including India, China, Malaysia, and Laos [16–20], stressing the need for context-specific research in Cambodia.

This study aims to build an extended cascade of HTN care in Cambodia and explore the demographic characteristics explaining the ‘drop-out’ in the CoC. The extended cascade is composed of six bars: prevalence (i), screening (ii), diagnosis (iii), treatment in the last 12 months (iv), treatment in the last three months (v), and being under control (vi). The analysis will inform policymakers about the health system’s performance and differences in risk characteristics across the continuum of HTN care. Additionally, it will provide insights into the reasons for inequities in access and outcomes.

## Methods

### Study design and setting

The current study employs data from a cross-sectional, population-based household survey. This survey was set up to assess the state of T2D and HTN care across Cambodia with a specific focus on three different interventions. The study was conducted in five purposively selected ODs with different combinations of the above-cited health system interventions to assess outcomes of the different interventions for HTN and T2D care in the population aged 40 years and older (Table 1).

**Table 1:** Selected ODs and health system interventions

**Province (OD Name)**	**Type of Health System Intervention**
Takeo (OD Daunkeo)	Hospital-based care (a) + Health center-based care (b) + Community-based care (c) + private care
Kampong Speu (OD Kong Pisei)	Community-based care (c) + private care
Prey Veng (OD Pearaing)	Hospital-based care (a) + Health center-based care (b) + private care
Siem Reap (OD Sort Nikum)	Hospital-based care (a) + Health center-based care (b) + private care
Oddar Meanchey (OD Samrong)	Hospital-based care (a) + private care

### Sample size and sampling

The sample size of this study was calculated to allow for the prevalence estimated for the least prevalent of the two health conditions, T2D. The inputs for the calculation included a T2D prevalence of 10%, a margin of error of 0.01, and a 95% confidence interval (95% CI). The sample size then accounted for a 1.5 design effect, 20% non-response rate, and average household size of 4.6. As a result, the total estimated sample size was 5280 individuals. The survey was conducted from July–October 2020. This survey used a multi-stage stratified random cluster sampling design. First, five ODs were purposively selected. Second, 44 villages per OD were randomly selected using the p-proportional-to-size sampling method. Third, 24 households per village were selected using probability systematic sampling from the household list, which was composed of households with adults aged 40 years or older in the selected village, provided by the village chief.

**Fig. 1:**
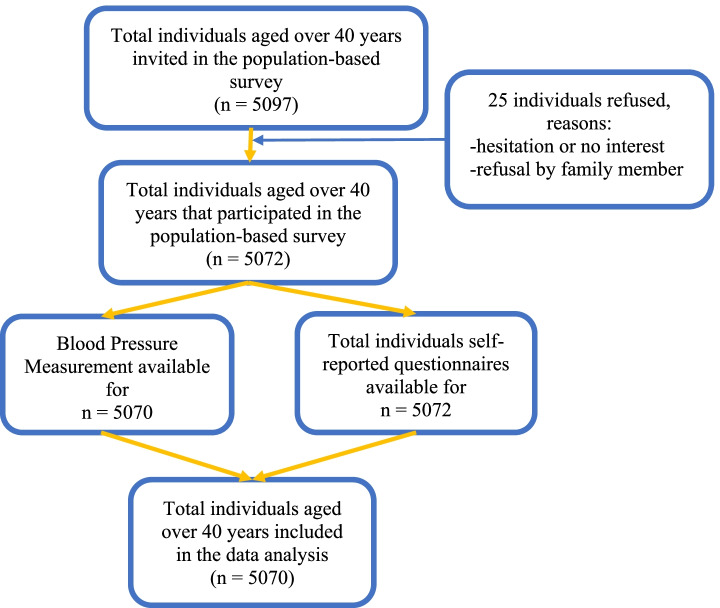
Flow chart of sample selection

Finally, one adult aged 40 years or older was randomly selected from each selected household. A total of 5097 eligible adults were invited for the study and were appointed to gather at the village chief’s house or pagoda in the morning on dates indicated in the invitation letters. We informed the participants about the time, location for the appointment, contact information, and other dietary requirements in each letter. They were asked to fast at night before the appointment date and not drink or eat before coming to the appointment sites. Two repeated call-backs were applied if the selected adult did not present. If the invited participant did not show up at the appointment site even after these two call-backs, a replacement method was used by replacing the selected household with the subsequent household in the list. Out of the 5097 people invited, 5072 participated, resulting in a 99.5% response rate. After excluding two records with missing information on BP measurement, 5070 participants were included in the analyses (Fig. 1).

### Measurements and data collection

#### Measurements

The BP was measured by trained data collectors using an Omron Automatic Blood Pressure Monitor (Model JPN500, Japan). Before the measurement, all participants rested for five minutes. When subjects were ready to have their BP taken, they were asked to keep calm and not think about stressful things. Then, they were asked to take off excess clothing because that might impede the BP monitor or limit the blood flow. After that, they were asked to sit comfortably for five minutes with their backs resting against a chair and legs and ankles uncrossed. Three systolic and diastolic BP measurements were recorded and taken after 5–15 min of rest. The average of the second and third measurements was used for the analysis. This procedure comprises high validity since it has been adopted from the WHO STEPS Survey protocol [21].

#### Elements of cascade of HTN care

The cascade of HTN care is composed of six bars, and each bar shows the step in the HTN continuum of care. The CoC in this study has been constructed using a fixed denominator (all respondents aged over 40 years having HTN) employing a conditional approach. The details of how each bar is constructed can be seen in Table 2.

**Table 2:** Elements and Construction of Cascade of HTN Care using a fixed denominator conditional approach

Bar of the Cascade	Definition/nominator	Condition
Bar 1: Prevalence	Respondents with survey-measured SBP ≥ 140 mm Hg and/or DBP ≥ 90 mm Hg or respondent self-reporting as having been told by healthcare professionals that they have HTN [7, 22]	All respondents aged over 40 years with a biochemical measurement at time of the survey
Bar 2: Screen	Respondents with raised BP or having HTN reported that they had their BP measured in the last 3 years [23]	All respondents aged over 40 years had HTN
Bar 3: Diagnosis	Respondents with a history of having screened for HTN have had their HTN diagnosed by a healthcare professional (at either public or private health facility) [23]	All respondents with raised BP or having HTN reported that they had their BP measured in the last 3 years
Bar 4: Treatment in the last 12 months	Respondents with the confirmed diagnosis have received treatment for their HTN in the last 12 months [23]	All respondents with a history of having screened for HTN have had their HTN diagnosed by a healthcare professional (at either public or private health facility)
Bar 5: Treatment in the last 3 months	Respondents with treatment history in the last 12 months have received HTN treatment in the last 3 months [23]	All respondents with the confirmed diagnosis have received treatment for their HTN in the last 12 months
Bar 6: Under-Control	Respondents with treatment history in the last 3 months have their SBP < 140 mm Hg and DBP < 90 mm Hg [7, 22]	All respondents with treatment history in the last 12 months have received HTN treatment in the last 3 months

*SBP* Systolic Blood Pressure, *DBP* Diastolic Blood Pressure, *Hg* Mercury

#### Correlates of the HTN cascade of care

To explore the characteristics explaining the ‘drop-out’ in the CoC, variables that were shown to be associated with different HTN care outcomes in previous literatures were included [24–27].

The socio-demographic variables included gender (male, female), age group (40–49, 50–59, over 60 years), level of education (no education, some primary or completed primary education, some or completed secondary or higher education), marital status (married or living together, divorced or widowed, single), occupation (unemployed or housewives or retired, non-agricultural work, agricultural work) and household size (1–3 members, 4–5 members, more than 6 members).

Socio-economic factors include wealth quintile (poorest, poor, medium, rich, richest) and covered by insurance (no, yes if covered by either Health Equity Fund (HEF) card or National Social Security Fund (NSSF) card).

There is a specific transition for each bar of the cascade, so additional correlates were included specifically where relevant from one bar to another. Disease-related variables such as disease duration (less than 3 years, 3–5 years, more than 5 years), the occurrence of complications (no, yes if a doctor has ever told them that they have eye problems or kidney problems or lost sensation of peripheral membranes), and information of healthcare organization such as type of health facility (public health facility, private health facility, public and private, don’t know) were used to predict the continuation from diagnosis (bar 3) to treatment in the last 12 months (bar 4). The type of health facility attended at the time of diagnosis was used as a predictor of dropping out of treatment in the last 12 months (bar 4) and treatment in the last three months (bar 5). Also, the type of health facility attended to receive treatment in the last three months was used as a predictor of achieving BP under control (bar 6). Additionally, since this study aimed to assess the continuum of care in five purposively selected ODs (Daunkeo, Kong Pisei, Pearaing, Sort Nikum, Samrong) with different healthcare interventions (Table 1), ‘OD’ was included as a control variable.

#### Data analysis

**Fig. 2:**
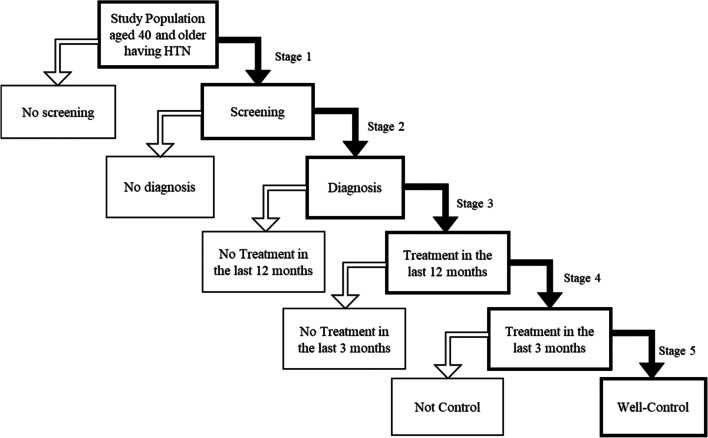
Model illustrating different stages of the logistic regression

Descriptive statistics were used to generate the prevalence of each bar of the cascade. The cascade was built with a fixed denominator employing a conditional approach (Table 2) and the regression is based upon a non-fixed denominator employing a conditional approach (Fig. 2). Logistic regression was also used to determine the profile of those who dropped out from each cascade bar. All variables were retained in the regression regardless of their significant status in the bivariate analysis. Data analyses were done using STATA version 14 (StataCorp. 2015. Stata Statistical Software: Release 14. College Station, TX). Weights were accounted for in all analyses.

We conducted the logistic regression using different sub-samples at each stage of the cascade:

- Stage 1: assessed the characteristics associated with HTN screening (Code 1 if respondents with raised BP or having HTN reported screening in the last three years and 0 if respondents with raised BP or having HTN but no screening).
- Stage 2: assessed the characteristics associated with being diagnosed (Code 1 if respondents received screening and had their HTN diagnosed by a healthcare professional and 0 if respondents receive screening but no diagnosis).
- Stage 3: assessed the characteristics associated with receiving treatment in the last 12 months (Code 1 if respondents received diagnosis and treatment in the last 12 months and 0 if received diagnosis but no treatment in the last 12 months).
- Stage 4: assessed the characteristics associated with receiving follow-up treatment in the last three months (Code 1 if respondents received treatment in the last 12 months and keep going for follow-up treatment in the last three months, and 0 if received treatment in the last 12 months and not going for follow-up treatment in the last three months).
- Stage 5: assessed the characteristics of achieving well-controlled HTN (Code 1 if respondents received treatment in the last three months and have BP < 140/90 mm Hg, and 0 if received treatment in the last three months but BP ≥ 140/90 mm Hg).

The regression was conducted only where the loss was greater than 5%.

## Results

### Socio-demographic characteristics of the study participants

Most participants were female (61.7%), aged over 60 years (38.4%), had an educational background of some primary or completed primary education (13.5%), were agricultural workers (61.2%), were married or living together (76.4%), and had no insurance card (social protection scheme whether HEF or NSSF card) (79.6%). There was an approximately homogenous proportion of each level of the wealth quintile and category of household size (Table 3). Of those with HTN, the majority were aged over 60 years (44.6%), had an educational background of some or completed secondary or higher education (39.2%), were unemployed or retired or staying at home as housewives (45.9%), and were divorced or widowed (41.1%). There was a fair proportion of HTN among males vs. females, between ODs, for each level of the wealth quintile, and each category of household size (Table 3).

**Table 3:** Socio-demographic characteristics of the study participants

Socio-Demographic Characteristics	Among all study participants (*n* = 5070)		Among people with hypertension (*n* = 1729)	
	**Unweighted Frequency (n)**	**Weighted Percentage (%)**	**Unweighted Frequency (n)**	**Weighted Percentage (%)**
**Gender**				
Male	1867	38.3	594	34.6
Female	3203	61.7	1135	35.5
**Age (years)**				
40–49	1331	26.8	299	22.4
50–59	1748	34.8	558	34.6
Over 60	1991	38.4	872	44.6
**Education**				
No education	1528	28.8	536	35.3
Some primary or completed primary education	2870	57.7	952	34.1
Some or completed secondary or higher education	672	13.5	241	39.2
**Occupation (main occupation)**				
Unemployed or housewives or retired	1036	19.6	470	45.9
Non-agricultural sector	951	19.1	369	40.9
Agricultural sector	3083	61.2	890	29.8
**Wealth Quintile**				
Poorest	1011	15.8	339	33.0
Poor	1013	19.4	313	31.8
Medium	1018	20.3	332	35.1
Rich	1013	21.3	367	36.4
Richest	1015	23.1	378	38.4
**Marital Status**				
Married or living together	3492	76.4	1120	33.9
Divorced or widowed	1477	21.2	581	41.1
Single	101	2.4	28	22.9
**OD**				
Daunkeo	1019	14.2	352	34.5
Kong Pisei	1017	32.5	345	36.1
Pearaing	1025	13.3	360	35.1
Sort Nikum	1005	23.2	347	34.8
Samrong	1004	16.7	325	34.3
**Household Size**				
1–3 members	1598	27.8	565	35.3
4–5 members	2059	39.4	681	34.8
more than 6 members	1413	32.8	483	35.5
**Insurance**				
No	3900	79.6	1326	34.5
Yes (any insurance card)	1170	20.4	403	37.6

### Cascade of Hypertension Care

**Fig. 3:**
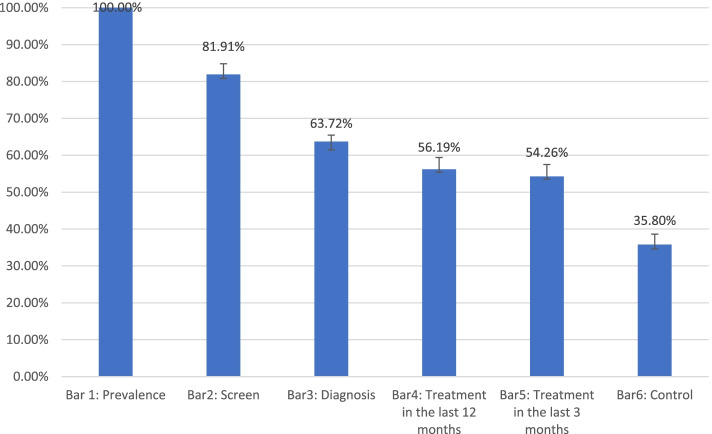
Cascade of hypertension care with fixed denominator conditional approach

Figure 3 provides an overview of the extended cascade with six bars. The prevalence of HTN (i) among study participants was 35.2%, of which 81.9% had their BP measured in the last three years (ii). Over 63% of those receiving screening were diagnosed by a healthcare professional as hypertensive patients (iii). Among these hypertensive patients, 56.2% had received treatment in the last 12 months (iv), and 54.2% had received the follow-up treatment in the last three months (v). The results showed that only one out of three people (35.8%) with HTN had their BP under control (vi).

### Demographic characteristics explaining the dropping-out in the cascade

Table 4 outlines the risk characteristics explaining the dropping-out from each step of the cascade.

**Table 4:** Results from multivariate logistic regression explaining the drop-out in the CoC

Characteristics	Receive Screening if having raised BP or having HTN	Receive Diagnosis after being screened	Receive treatment in the last 12 months after being diagnosed	Receive follow-up treatment in the last 3 months after treatment in the last 12 months	Achieve Under-Control for hypertension
	(*n* = 1405)	(*n* = 1111)	(*n* = 958)	(*n* = 920)	(*n* = 607)
	**AOR**	**AOR**	**AOR**	**AOR**	**AOR**
**Gender**					
Male	1	1	1		1
Female	3.64*** (2.52–5.26)	3.67*** (2.05–5.41)	1.85* (1.06–3.26)		2.09*** (1.33–3.28)
**Age (years)**					
40–49	1	1	1		1
50–59	2.07** (1.34–3.19)	3.37*** (2.23–4.82)	3.68*** (1.86–7.29)		1.25 (0.62–2.51)
Over 60	3.67*** (2.32–5.79)	7.17*** (4.37–11.76)	2.78** (1.45–5.31)		1.41 (0.70–2.82)
**Education**					
No education	1	1	1		1
Some primary or completed primary education	0.86 (0.58–1.26)	0.98 (0.64–1.50)	0.89 (0.55–1.43)		1.18 (0.78–1.76)
Some or completed secondary or higher education	1.18 (0.68–2.04)	0.88 (0.48–1.60)	0.73 (0.28–1.94)		1.07 (0.54–2.16)
**Occupation (main occupation)**					
Unemployed/Housewives /retired	1.58 (0.96–2.59)	1.87** (1.10–3.16)	1.65 (0.96–2.86)		1.25 (0.80–1.95)
Non-agricultural sector	1.43 (0.94–2.18)	1.33 (0.84–2.12)	2.09** (1.18–3.68)		1.24 (0.77–1.99)
Agricultural sector	1	1	1		1
**Wealth Quintile**					
Poorest	1	1	1		1
Poor	1.88* (1.11–3.19)	1.18 (0.63–2.20)	1.42 (0.77–2.62)		1.16 (0.62–2.17)
Medium	1.77* (1.03–3.07)	0.91 (0.47–1.74)	1.53 (0.78–3.03)		2.22* (1.15–4.30)
Rich	2.14* (1.17–3.91)	0.74 (0.39–1.40)	4.27*** (1.85–9.84)		1.35 (0.72–2.88)
Richest	2.39** (1.32–4.32)	0.56 (0.31–1.03)	3.95** (1.74–8.97)		1.50 (0.78–2.88)
**Marital Status**					
Married or living together	3.62* (1.23–10.63)	0.43 (0.07–2.44)	3.51 (0.71–17.27)		0.47 (0.08–2.53)
Divorced or widowed	4.72** (1.60–13.92)	0.28 (0.05–1.65)	4.06 (0.84–19.68)		0.50 (0.09–2.68)
Single	1	1	1		1
**Operational District (OD)**					
Daunkeo	0.96 (0.57–1.60)	0.82 (0.47–1.44)	0.52 (0.26–1.06)		0.65 (0.35–1.20)
Kong Pisei	1.57 (0.92–2.70)	0.94 (0.56–1.59)	1.03 (0.51–2.11)		0.52* (0.29–0.92)
Pearaing	1.07 (0.61–1.90)	1.28 (0.72–2.27)	0.79 (0.38–1.65)		0.70 (0.37–1.28)
Sort Nikum	1.03 (0.63–1.70)	1.24 (0.72–2.14)	1.22 (0.59–2.49)		0.51* (0.29–0.89)
Samrong	1	1	1		1
**Household Size**					
1–3 members	1	1	1		1
4–5 members	0.76 (0.51–1.12)	1.12 (0.74–1.69)	0.91 (0.54–1.54)		0.87 (0.57–1.33)
more than 6 members	0.93 (0.59–1.45)	1.18 (0.76–1.84)	1.12 (0.63–1.98)		0.83 (0.51–1.34)
**Insurance**					
No	1	1	1		1
Yes (any insurance card)	1.00 (0.64–1.56)	1.08 (0.66–1.74)	0.86 (0.52–1.41)		1.05 (0.64–1.72)
**Complication**					
No			1		1
Yes (have any complication)			1.14 (0.73–1.77)		0.74 (0.51–1.07)
**Disease Duration**					
Less than 3 years			1		1
3–5 years			1.20 (0.68–2.10)		0.74 (0.48–1.13)
More than 5 years			0.69 (0.40–1.21)		0.56* (0.35–0.89)
**Health Facility for Treatment after being diagnosed**					
Public			1		
Private			0.80 (0.49–1.29)		
Public and Private			0.44 (0.03–6.00)		
Non-Health Professional			0.046 (0.01–0.33)		
**Health Facility for Treatment in the last three months**					
Public					1
Private					0.58* (0.37–0.91)
Public and Private					0.82 (0.26–2.56)
Non-Health Professional					//

^*^ *p* ≤ 0.05, ** *p* ≤ 0.01, *** *p* ≤ 0.001

### Characteristics associated with HTN screening

Women were more likely to receive screening than men (Adjusted Odds Ratio (AOR) = 3.64, 95% CI = 2.52–5.26). People aged 50–59 years (AOR = 2.07, 95% CI = 1.34–3.19) and more than 60 years (AOR = 3.67, 95% CI = 2.32–5.79) had greater odds of receiving screening than those aged 40–49 years. People from poor households (AOR = 1.88, 95% CI = 1.11–3.19), medium wealth households (AOR = 1.77, 95% CI = 1.03–3.07), rich households (AOR = 2.14, 95% CI = 1.17–3.91), and richest households (AOR = 2.39, 95% CI = 1.32–4.32) had higher odds of receiving screening than those from the poorest households. People who were married or living together (AOR = 3.62, 95% CI = 1.23–10.63), and divorced or widowed people (AOR = 4.72, 95% CI = 1.60–13.92) were more likely to receive screening than single people.

### Characteristics associated with being diagnosed

Out of people who had been screened for HTN, female respondents were more likely (AOR = 3.67, 95% CI = 2.05–5.41) to receive a diagnosis than male respondents. The odds of receiving a diagnosis were greater for people aged 50–59 years (AOR = 3.37, 95% CI = 2.23–4.82) and more than 60 years (AOR = 7.17, 95% CI = 4.37–11.76) than those aged 40–49 years. People who were unemployed or had retired (AOR = 1.87, 95% CI = 1.10–3.16) were more likely to receive a diagnosis than those who worked in the agricultural sector.

### Characteristics associated with receiving treatment in the last 12 months

Out of people who were diagnosed with HTN, women were more likely to receive treatment in the last 12 months than men (AOR = 1.85, 95% CI = 1.06–3.26). Within this group, the odds of receiving treatment in the last 12 months were greater for people aged 50–59 years (AOR = 3.68, 95% CI = 1.86–7.29) and more than 60 years (AOR = 2.78, 95% CI = 1.45–5.31) than those aged 40–49 years. People who worked in non-agricultural sectors (AOR = 2.09, 95% CI = 1.18–3.68) were more likely to receive treatment in the last 12 months than those who worked in the agricultural sectors. People from rich households (AOR = 4.27, 95% CI = 1.85–9.84) and richest households (AOR = 3.95, 95% CI = 1.74–8.97) had higher odds of receiving treatment in the last 12 months than those from poor households.

### Characteristics associated with receiving follow-up treatment in the last three months

There was a small drop from those people who received treatment in the last 12 months (*n* = 958) to receiving follow-up treatment in the last three months (*n* = 920); therefore, regression analysis was not performed on this part.

### Characteristics associated with achieving well-controlled hypertension

Out of people who were treated for HTN in the last three months, female patients (AOR = 2.09, 95% CI = 1.33–3.28) were more likely to get their HTN under control than male patients. The odds of achieving HTN under control was higher for people from medium wealth households (AOR = 2.22, 95% CI = 1.15–4.30) than those from poor households. The odds of achieving good control of HTN were greater in OD Samrong than OD Kong Pisei (AOR = 0.52, 95% CI = 0.29–0.92) and OD Sort Nikum (AOR = 0.51, 95% CI = 0.29–0.89). The odds of getting HTN under control were greater for people living with HTN less than three years than those more than five years (AOR = 0.56, 95% CI = 0.35–0.89) and for people using public healthcare services than those using private healthcare services (AOR = 0.58, 95% CI = 0.37–0.91).

## Discussion

Our results indicate that people drop out across all steps of the cascade, with the most substantial losses in the steps from having HTN to screening, from screening to diagnosis, and from treatment in the last three months to having one’s HTN under control. Around 55% of those diagnosed with HTN have a good continuation of their care process, from in treatment to a regular follow-up. Only one-third of the study participants with HTN reached the last bar of the cascade of care—well-controlled. This shows that incorporating HTN as basic care combined with the multitude of additional interventions has not sufficiently addressed this public health problem in Cambodia.

Overall, the produced cascade of HTN care is comparable; however, there are some marked differences with those produced by similar studies in the region. Compared to the study on adults aged over 40 years in Indonesia, the prevalence of HTN in Cambodia was lower (47.8% in Indonesia vs. 35.1% in Cambodia), whereas the proportions of people aware of their diagnosis (29.4% vs. 63.7%), under-treatment (25% vs. 54.3%), and control (9.0% vs. 35.8%) were higher [27]. When compared to the results from a previous study in central Vietnam in 2015 among the population aged 40–69 years, our study had a lower prevalence (44.8% in Vietnam vs. 35.1% in Cambodia), a comparable proportion of awareness of diagnosis (67.3% vs. 63.7%), a higher proportion in treatment (33.2% vs. 54.2%), and a higher proportion in control (12.2% vs. 35.8%) [26]. Another study in the rural community people in Thailand on the population aged 35–60 years produced a similar pattern only in the proportion of awareness and treatment, but higher result in control [28]. Possible explanations for the lower prevalence but a higher number of people achieving control in Cambodia compared to the studies in central Vietnam and Indonesia, and the similar results compared to rural Thailand might be explained by the population characteristics and the resources and priority-setting in each setting, for instance, the coverage of HTN care, the performance in health service delivery, and population lifestyle.

The produced CoC in this study provides crucial evidence to the Cambodian government to steer their strategies for HTN control. The CoC approach clearly necessitates that the National Strategy needs to recognize the importance of ensuring continuous service use along the cascade of HTN care in order to durably improve HTN care in Cambodia. The CoC methodology applied in this study could be a valuable tool to monitor progress towards reaching this continuum of care.

The three largest drops demonstrated by our study provide insight on the gaps in the health system in HTN care; policymakers must take action regarding these to prevent a failure in the health system. First, a substantial loss of people living with HTN who reported not receiving screening suggests the importance of making screening programs more widely available at the primary healthcare level by increasing coverage and capacity of HCs in providing NCD service delivery. Second, the biggest drop from screening to diagnosis sheds light on the importance of on-time detection. This detection is vital since early detection can minimize the long-term impact on the health system and economic development [29, 30].

The last biggest loss was from people who received follow-up treatment in the last three months to people who had their HTN under control, this implies inadequate treatment. Despite having been on anti-hypertensive treatment, a large proportion of people could not achieve optimal BP control. This problem requires a combination of quality treatment and comprehensive intervention of lifestyle modification.

The findings of our study on the profiles of those dropping out from one bar of the cascade to another also demonstrated that, compared to the poorest households, people from the poor to richest households were more likely to receive screening, but only people from rich households remained in treatment and those from medium wealth households achieved good control of HTN. Socio-economic status impacted the utilization of health services as mentioned in many published studies [31–33]. In our study, most of the rich population reported using care from private facilities while at the same time it was demonstrated that those visiting private facilities were less likely to have their HTN controlled. This results in the fact that, even though people from the richest households were more likely to be in care, they were not more likely to have their HTN under control compared to people from the poorest households who were in treatment. This reflects the inefficiency of care and treatment which restrains users from achieving the optimal goal of care. The role of private healthcare is undeniable for HTN care when a majority of Cambodians have been using private care. WHO reports on the relative efficiency of public and private service delivery showed inconclusive evidence due to lack of data and absence of evidence-based study on efficiency [34]. To ensure the most efficient HTN health service delivery, a mixed strategy is needed from the government by introducing measurement of efficiency to identify key causes and constraints to improve the performance of HTN healthcare nationwide. Additionally, introducing innovative financial schemes to cover HTN service uses can help keep patients in the continuum of care without the risk of catastrophic health expenditure regardless of the type of health facility they use.

The inequities in the continuum of HTN care between men vs. women, population under or equal to 49 years vs. over 50 years, people living with HTN less than five years vs. more than five years, and agricultural vs. non-agricultural workers demonstrate that the health system is not reaching all people equally and/or is not producing equal outcomes across these different groups. This result is not affected by the large proportion of female participants in our survey. Evidently, the prevalence of HTN is not so different between males and females (34.6% vs. 35.5%) and the results of the 2017 and 2019 Cambodia Socio-Economic Surveys found that women sought care at the health facility more than men in all areas in Cambodia [35, 36]. Men were found to have a lower control rate and delay in seeking help when they became ill [37, 38]. It is clear that an innovative health system approach is required to tackle these issues. In light of the limited resources for health in Cambodia, one potential pathway to produce more equitable access to essential HTN care is the double strategy of building additional community-based care (to improve access) while improving the quality of facility-based care (to improve health outcomes). Taking into account the required continuum of care for chronic conditions such as HTN, community care could be a valuable extension of the professional healthcare team, by providing social support in medication adherence, self-management support through home visits, referral for facility-based care, reporting, and follow-up. This approach has been successfully used to improve management and reduce the gap of inequities of HTN care in Nepal [39] and Pakistan [40].

### Strengths and limitations of the study

This study is subject to some limitations. Firstly, based on the standard definition, the diagnosis of HTN should have been based on two or more BP measurements taken at two or more visits after the initial screening [2]. In our study, three BP measurements were taken during a single visit, so the prevalence might be over-estimated, and the control might be under-estimated. However, we have applied the standardization of the WHO STEP approach [21] and analyses to overcome this problem. Secondly, our study was a cross-sectional study, so causal interpretations are not possible. Thirdly, it is very difficult to assess the net impact of the interventions, as a large proportion of HTN and T2D care is provided by private facilities in each OD. However, the strength of this study is the large sample including both self-reported questions and standardized BP measurement. Lastly, the purposively selected five ODs limited the generalizability of this study; however, the rigorous sampling procedure at both village and household levels reflected a situation of cascade of HTN care in Cambodia. A comprehensive overview of the extended cascade of HTN care to outline population-level estimates of patients that make it through each stage of the cascade is guaranteed.

## Conclusion

Our study found a high prevalence of HTN (35.2%) among adults aged 40 years and older while only one-third of those with HTN achieved a complete continuum of care and good health outcome. Our findings demonstrated that the CoC approach can be a useful tool to assess the effectiveness of HTN care and associated health outcome and should be used as a monitoring tool to assess the national strategy and potentially advise investments in healthcare for chronic conditions as it provides insights into where (as well as which) people are along the continuum of HTN care. Priority (for interventions and investments in expanding HTN care) should be given to males, those aged 40–49 years, and poorer population groups. This will not only allow achieving the best outcome but also reduce health inequities, thereby contributing to achieving Universal Health Coverage. Among people receiving HTN care in the last three months, those accessing care in private facilities were less likely to achieve well-controlled BP. These results demonstrated that a double strategy of quality health facility-based and community-based interventions is needed at different levels to increase screening uptake, awareness of their diagnosis, continuous treatment, and good control of their HTN. To minimize the inequitable access to healthcare, the interventions should use the data produced by the CoC approach to focus on the major drops and those likely to drop in the cascade.

With political commitment towards Universal Health Coverage, this study took the first step by providing a comprehensive overview of the performance of current HTN care along with all steps of the continuum of care and an in-depth understanding of the characteristics of the people at risk of dropping out at each step of this continuum. This is indeed essential to see how far Cambodia is in terms of HTN care to reach Universal Health Coverage. Future research should be conducted to closely look at the healthcare performance for both public and private care nationwide.

## Data Availability

The datasets generated and/or analysed during the current study are not yet publicly available (because the main impact manuscript has not yet been published), but are available from Prof. Edwin Wouters (edwin.wouters@uantwerpen.be) and Asso. Prof. Por Ir (ipor@niph.org.kh) on reasonable request.

## Abbreviations

HTN: Hypertension
CVD: Cardiovascular Diseases
HCs: Health Centers
RHs: Referral Hospitals
NCD: Non-Communicable Disease
WHO PEN: World Health Organization Package of Essential Non-Communicable Disease Interventions
MoH: Ministry of Health
T2D: Type 2 Diabetes
ODs: Operational Districts
CoC: Cascade of Care
BP: Blood Pressure
SBP: Systolic Blood Pressure
DBP: Diastolic Blood Pressure
Hg: Mercury
HEF: Health Equity Fund
NSSF: National Social Security Fund
AOR: Adjusted Odds Ratio
95% CI: 95% Confidence Interval
